# Effects of Aging Attitudes on Development in Old Age

**DOI:** 10.1093/geroni/igab046.726

**Published:** 2021-12-17

**Authors:** Maria Clara P de Paula Couto, Klaus Rothermund

## Abstract

This session will focus on aging attitudes and their effects on different aspects of development in old age (e.g., preparation, age stereotypes, age discrimination, and well-being). Cultural differences and how they shape individual aging are also explored. The first two presentations focus on cross-cultural differences in preparation for old-age. Nikitin et al. examine financial preparation and how expectations about support from the state influence it. People’s beliefs about the utility and the risk of aging preparation and their role in preparatory activities is investigated by Kim-Knauss et al. Tsang et al. explore age differences in pursuing autonomy and independence during the COVID-19 pandemic and the role of perceived social obligation. Cultural differences in the accuracy between perceived retrospective changes in well-being and actual changes is explored by Park et al. The last presentation (de Paula Couto et al.) focuses on country- and age-related differences in personal experiences of age discrimination in different life domains. Taken together, findings suggest that attitudes toward, and preparation for aging, are not static. Situational contexts and personal assessments of the contexts can shape such attitudes and behavior.

